# A Systematic Review of Recent Studies on Hospital Readmissions of Patients With Diabetes

**DOI:** 10.7759/cureus.67513

**Published:** 2024-08-22

**Authors:** Ruchi D Kukde, Aindrila Chakraborty, Jaymeen Shah

**Affiliations:** 1 Department of Organization, Workforce, and Leadership Studies, Texas State University, San Marcos, USA; 2 Department of Information Systems and Analytics, Texas State University, San Marcos, USA

**Keywords:** systematic review, prediction, diabetes, risk factors, hospital readmissions

## Abstract

Hospital readmissions are a major area of concern across the healthcare ecosystem. Diabetes mellitus (DM) and associated complications significantly contributed to hospital readmissions in 2018, placing it among the leading causes alongside septicemia and heart failure. Diabetes is an urgent public health concern that has reached epidemic proportions globally. Compared to the early 2000s, the prevalence of diabetes among individuals aged 20-79 years in the US has significantly increased. This research provides an in-depth examination of diabetes-related hospital readmissions and reviews recent studies (2015-2023) to understand the characteristics, risk factors, and potential outcomes for re-admitted diabetes patients. The study identified 21 articles that met the inclusion criteria to provide valuable insights and analyze risk factors associated with these readmissions. The findings indicated that risk factors such as age, demographics, income, insurance type, severity of illness, and comorbidities among diabetic patients were critical and warranted further investigation. Diabetes awareness, quality of hospital care, involvement of healthcare providers, timely screening, and lifestyle changes were noted as important factors to improve the effectiveness of healthcare delivery, reduce diabetes-related complications, and eventually lower preventable hospital readmissions.

## Introduction and background

Hospital readmission is defined as the re-hospitalization or hospital visit of a patient after discharge within a specific period to the same or different medical institution after the previous visit [1]. Hospitalization indicates the severity of the ailment and presents an opportunity to support patients with coordinated care and observation in a facility. On the other hand, re-hospitalization has been frequently considered as an indicator of the failure of caregiving, either in transferring the patient from one medical or care-providing institution to another or between the facility and home, in a certain period, e.g., 30 days [2]. Hospital revisits or readmissions generally imply incomplete treatments, medication errors, negligence from healthcare service providers (e.g., doctors, nurses), or unsuccessful procedures during prior inpatient visits [1]. Due to the complex and intricate nature of the reasons resulting in hospital readmissions, the systematic investigation and in-depth analysis of hospital readmissions have been integral parts of US healthcare systems, with ‘hospital readmission’ being defined as a metric for research studies [3,4].

The impact of hospital readmissions is manifold; two of these aspects stand out in particular: financial burden and emotional toll on patients’ health [5]. A series of successive hospital visits and admissions has been reported to result in huge costs for patients and healthcare systems [3]. For instance, it was reported that the cost of hospital readmissions incurred by Medicare was estimated to result in a massive annual cost of USD 26 billion [6]. Addressing the emotional cost of readmissions, it was noticed, quite unfortunately, how rarely the discussions with patients and caregivers encompassed this issue. One of the studies emphasized patients’ belief in preventable readmissions and underlined the need for healthcare providers to recognize their role and address discharge timing, home health, follow-up, and skilled services to alleviate patient frustration [2]. To tackle the challenges of hospital readmissions and mitigate the socioeconomic and emotional costs incurred by hospitals, physicians, payers, and healthcare providers, the US federal government introduced national initiatives such as the Centers for Medicare & Medicaid Services (CMS) Hospital Readmissions Reduction Program (HRRP) [7]. The HRRP initiative, based on the pay-for-performance policy, aims to reduce avoidable readmissions by improving communication, care coordination, and discharge plans [8].

Diabetes-related hospitalizations

Diabetes continues to be an urgent public health issue affecting millions of individuals throughout the world, with contributing factors attributed to an aging population, longer life expectancy, a sedentary lifestyle, and increasing obesity. According to the International Diabetes Federation (IDF) 2021 reports, a total of 1.2 million children and adolescents (age group: 0-19 years) and 537 million adults (age group: 20-79 years) were estimated to be living with type 1 and type 2 diabetes mellitus (DM) in 2021, respectively [9]. The prevalence of gestational diabetes was estimated to be 16.7% [9]. In the United States, the frequency of DM increased at a faster rate compared to that of chronic conditions such as heart disease, cancer, and respiratory illnesses [4]. Over eight million diabetes patients were discharged from hospitals in the US in 2018, accounting for about 30% of all discharges. Diabetes mellitus with complications was third among the top 20 principal diagnoses associated with 30-day re-hospitalizations of adults in the US [10]. In 2018, approximately 10.5% of the US population had diabetes, and 32% of readmissions were triggered by diabetes [10]. With an average length of stay of 4.66 days, the aggregate costs incurred by hospitals during 2018 were in the vicinity of USD 12.8 billion for patients hospitalized with endocrinological complications, including DM, raising serious concerns about the rising prevalence of diabetes and associated hospitalization costs in the country. Given these alarming statistics, it was important to study and analyze the risk of readmissions for diabetes patients.

Diabetes care and readmissions: review of factors, risks, and strategies

A detailed study of hospital readmissions was important for understanding readmission risks and disease management techniques. Studies reported interconnected dynamic relationships between diabetes care aspects and hospital readmissions, and efforts to improve the quality of inpatient care to lower readmission rates were found to be likely to eventually reduce healthcare costs [11]. It was noted that lower socioeconomic status, racial/ethnic minority status, comorbidities, insurance type, emergent or unplanned admission, and prior hospitalization were some of the risk factors for readmissions [11]. Researchers identified hospitals catering to low-income patients and hospitals with inadequate care facilities as major reasons for readmissions [12]. Consequently, it was argued that structured diabetes care and follow-up plans could reduce the likelihood of readmission or, in the event thereof, lower the length of stay [13]. Social determinants of health, including the socioeconomic conditions of patients, were highlighted, thus emphasizing the need for a greater focus on diabetes prevention and risk management [14].

In addition to socioeconomic status, researchers found that age, gender, and ethnicity, all of which were beyond the patient’s control, contributed to the increased risk of diabetes [4]. Based on age statistics, the percentage of adults with diabetes increased with age, reaching 29.2% for 65+ adults in 2019 [4]. Studies have reported that diabetes education, prevention, and care need to be tailored differently for men and women [15]. Among adults 18 years and older, the percentage of diagnosed diabetes was highest (17.8%) among White adults, compared to Black, Asian, and Hispanic individuals [4]. Patients’ lifestyles were found to be a major influencer of their diseased state in several ways. Statistical evidence in 2015-2018 demonstrated that tobacco users, smokers, overweight and obese individuals, and physically inactive persons were at higher risk of diabetes [4,16]. Studies showed that the availability of support systems had a positive impact on the ability of the diabetic patient to make long-term changes associated with the diabetes condition [17]. The use of self-management skills with proper education and awareness was also found to be conducive to regulating blood glucose levels [1,4,18].

Review of predictive analytics for assessing readmission risks

Researchers employed several quantitative methods, including predictive analytic models, which enabled healthcare providers to deliver better treatment, lower patient readmission rates, reduce readmission-related medical costs, and deliver better post-discharge strategies [1]. Literature studies have indicated that predictive models should possess several qualities, including high predictive ability, reliable and clinically relevant data, rigorous performance metrics, validation in populations where they are intended to be used, and generalizability to heterogeneous populations [19,20]. Due to the complex nature of diseases, many predictive models were effective under certain circumstances and ineffective when applied to different sets of electronic health records [21]. A recent review of different prediction models suggested that using electronic health records showed better predictive performance measures compared to the administrative data [21]. For predicting readmissions, data imbalance, data locality, data variety, data complexity, and model interpretability were identified as some of the major challenges [1,22-24].

An association among health information technologies, patient demographics, hospital characteristics, payer information, and patient readmission was reported [25]. Medical professionals, hospitals, and management benefitted from understanding how readmission risks were predicted. Regression-based prediction models were commonly employed for case studies pertaining to hospital readmissions of patients [26-28]. Various machine learning approaches for data analysis, pre-processing, and modeling were also reported for predicting readmissions [29-31]. While most of the studies focused on one instance of readmission, it was observed that individuals with diabetes had been readmitted multiple times for complications resulting from the same issues [11,32].

Motivation

A major focus of healthcare analytics research has been centered around assessing risk factors for hospital readmissions of diabetic patients [1,33]. Despite the published research on diabetes-related readmissions, there is a paucity of comprehensive reviews addressing readmissions and the role of data analytics in assessing readmission risks. Existing reviews include the investigation of the effects of glycemic control [33], examination of risk factors through surveys of global datasets [34], and understanding of readmission risks in patients with type 1 and type 2 DM [35]. While these reviews present systematic dissemination of these readmissions and meta-analyses with notable outcomes to propose quality measures, some of them include fewer articles (less than 10), focus on comorbidities associated with diabetes, or refer to findings published up to 2018. Reviews focusing on electronic health records collected in the United States pertaining to diabetes are also crucial to address gaps that need further investigation. With this motivation in mind, the aim of this study was to provide an in-depth and up-to-date comprehensive review of recent literature to contribute to the existing body of knowledge.

Study objective

The objective of the study was to conduct a systematic review with meta-analysis and risk of bias assessment of research studies related to the readmissions of diabetic patients in the United States until 2023. The research explored various factors such as patient characteristics, chronic diseases, and social disparities, which were associated with readmissions. By examining recent articles (2015-2023), the review identified research gaps and potential directions for future studies. The review contributed to the understanding of the current literature and supported initiatives designed to decrease the risk of readmission. Furthermore, the study underlined the need for targeted education for diabetes management. It explored the importance of early diabetes identification and implementation of management strategies to reduce readmissions. Keeping these considerations in view, the objective of this review paper was to analyze recent research and analytical methods used for predicting and assessing readmissions, identifying limitations, and suggesting future directions for research.

## Review

Materials and methods

In this study, Preferred Reporting Items for Systematic Reviews and Meta-Analyses (PRISMA) guidelines were used to conduct the systematic review [36]. Through a systematic search, research was carried out in the field of endocrinology, particularly measuring the effects and complications of diabetes, and identifying hospital readmissions. It focused on determining the risk factors, causes, and predictors involved in the readmissions for diabetes patients. The research articles identified through the PRISMA protocol were scrutinized and compared to extract methods, limitations, findings, and implications.

A structured process using spreadsheets was employed by the authors (R.K., A.C., and J.S.) for a comprehensive search of the literature. To improve clarity and rigor for systematically understanding the data, the spreadsheets consisted of relevant studies where each record instance summarized one article and the columns consisted of relevant fields such as study design, outcome, predictors, and significant findings as reported in the studies. Using the university library, the databases searched included Science Citation Index Expanded, Scopus, Medical Literature Analysis and Retrieval System Online (MEDLINE) Complete, Gale Academic OneFile, Gale OneFile: Health and Medicine, and Springer Nature Journals. The literature search was restricted to full-text journal articles. The keywords used were ‘re-hospitalization’ or ‘readmission’ or ‘re-admittance’ as mandatory for the search engine in the title of the articles. These keywords were used in association with the terms ‘diabetes mellitus’ or ‘diabetes’ or ‘type 1 diabetes’ or ‘type 2 diabetes’ in the article title. It was observed that type 2 diabetes emerged as more significant in terms of severity as compared to type 1 diabetes and gestational diabetes.

Study design and data extraction

The initial search yielded 1,573 records for assessment. Out of these records, 403 records were removed as duplicates. The final search yielded 1,170 records, which were screened for the relevance of the subjects of the articles. The authors (R.K., A.C., and J.S.) screened the potential articles by further adding another search criterion. The articles under the umbrella of topics such as cardiovascular diseases, economic disparities, health aspects, health services research, and transitional care were excluded considering the primary objective of our review. Therefore, a total of 437 studies were removed. Based on the non-availability of full-text articles, 83 articles were removed from the process, leaving 650 articles for further screening. The three authors (R.K., A.C., and J.S.) independently screened the titles and abstracts of approximately one-third (217, 217, 216) of the articles as per the predefined eligibility criteria.

**Figure 1:**
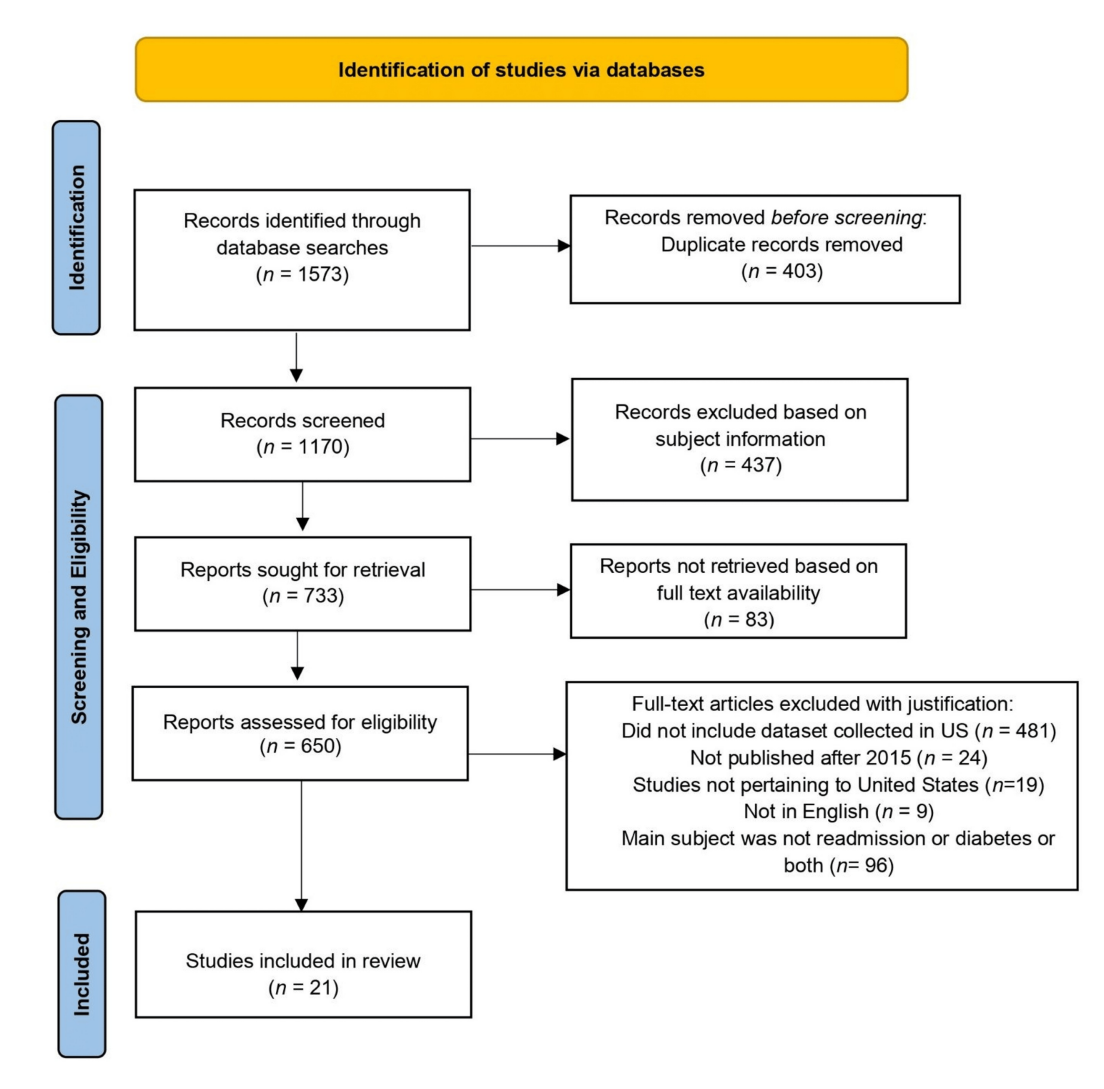
The PRISMA process of study selection PRISMA: Preferred Reporting Items for Systematic Reviews and Meta-Analyses

As per the study objective, we sought articles related to the examination of hospital readmissions in the United States. This involved restricting the search to articles published in the US or datasets related to hospitals in the US. A total of 500 studies were therefore excluded from the review. To restrict the full-text journal articles to those in the English language, the screening resulted in the further removal of nine studies in other languages. Keeping in view our intent to study recent literature, we included articles published in the most current period at the time of our study (2015-2023). This yielded 96 articles which were further assessed for eligibility. Figure 1 shows the flowchart of the PRISMA process used in this study [37]. A complete review of the papers was first carried out to understand the underlying studies with special emphasis on abstracts. Based on the latter, the three authors (R.K., A.C., and J.S.) independently and inductively assessed one-third of the articles for primary article purposes. By applying the PRISMA protocol, we were left with 21 studies for qualitative synthesis.

Data synthesis and reporting

Statistical analyses were performed using IBM SPSS Statistics software for Windows, version 28.0.1.0 (IBM Corp., Armonk, NY) [38]. Quantitative meta-analysis was performed to assess the relationship between patient demographics and socioeconomic variables, which were frequently observed in the studies [39]. Studies for meta-analysis were selected for inclusion based on predefined criteria as per the study objective. We employed random-effects models to calculate summary effect sizes. Effect sizes and confidence intervals were visually depicted using forest plots. In the analysis, I-squared statistics were used to assess heterogeneity among study findings. Statistical significance was set at a p-value <0.05. Owing to the diverse subsets of individuals with diabetes, the diversity of predictor variables, their sub-categories, and the nature of data collection in various datasets, a meta-analysis could be conducted for a few factors in this study. Additionally, this systematic review aimed at narrative summaries and the discussion of emergent themes from data synthesis.

Assessment of risk of bias

As per the study objective, research articles related to the prediction of risk factors for hospital readmissions were examined for risk of bias. Each study was assessed for risk of bias using the Prediction Model Risk of Bias Assessment Tool (PROBAST) [40,41] that included four domains: study participants, predictors, outcome, and analysis. Each domain was judged for risk of bias (low, unclear, high) using signaling questions [41]. Three studies were excluded from this assessment due to insufficient information or inapplicability of the tool [42-44]. Overall risk of bias assessment was also included in this study. Disagreements were resolved by discussion and reaching a consensus.

Results

Based on the PRISMA process, 21 studies were identified. These were studied thoroughly to understand their contributions, methods, similarities, and limitations.

Key Contributions and Methodologies

Table 1 presents the summary of key contributions of the selected articles to the field of research. Broadly, the selected studies encompassed a comprehensive examination of readmission risk and risk factors among individuals with diabetes. Key contributions included discussions on strategies like inpatient diabetes consultation, diabetes self-management education, and interventions. Additionally, the studies highlighted the importance of targeted nursing care improvement plans, preventive measures, and the exploration of socioeconomic factors and healthcare access. The methodologies employed in the studies were identified (Table 1). As per the inclusion criteria, articles focused on the prediction of readmissions of diabetic patients. The methods employed for analyses suggested that researchers focused on statistical methods, regression analysis in particular while exploring other approaches. In terms of the analysis methodologies, descriptive statistical analysis was noted in two articles [44,45]. The frequently used prediction model for readmissions was found to be multi-variable regression (17 out of 21 studies). Two studies used random forest (RF), naïve Bayes (NB), and decision tree ensemble methods [42,46].

**Table 1:** Key contributions and methodologies

Author, year	Key contributions to the field of research	Methodology
Rubin et al., 2023 [47]	Comparison of readmission risk and risk factors for adults with primary or secondary diabetes diagnosis; strategies include inpatient diabetes consultation for patients with a primary diagnosis of diabetes	Multivariable logistic regression
Timple and Kawar, 2022 [48]	Examination of predictors of type 2 diabetes mellitus (DM) with respect to comorbidities and endocrinology consultation; emphasis on diabetes self-management education; need for targeted plans to improve nursing care quality	Logistic regression
Shaka, 2022 [49]	Assessment of variation in readmissions in the pediatric population in terms of household income; necessary policies to provide accessibility to insulin and holistic healthcare irrespective of income	Multivariable logistic regression
Vasireddy et al., 2021 [50]	Analysis of trends and risk factors for unplanned readmissions of pediatric patients; prevention strategies: comprehensive care and discharge planning	Multivariable logistic regression
Thyagaturu et al., 2021 [51]	Analysis of heart failure-related admissions in the presence of DM Identification of high risk of readmissions to provide resources and education; preventive measures to optimize resource utilization	Multivariable Cox survival analysis and regression
Shaka et al., 2021 [52]	Description of characteristics of non-elective 30-day readmission among adult patients with diabetes; identification of predictors of readmission	Multivariable Cox regression
Shang et al., 2021 [46]	Implementation and comparison of machine learning (ML)-based readmission prediction methods; focus: identify patients who are at high risk of short-term readmission.	Random forest (RF), naïve Bayes (NB), decision tree ensemble methods
Smith et al., 2021 [53]	Explored the relationship between the timing of home healthcare initiation and 30-day rehospitalization risk	Multivariable logistic regression
Neto et al., 2021 [42]	Study of different scenarios for the prediction of hospital readmissions using ML; additional contextual features: eating habits, economic conditions, and insurance type	Data mining algorithms: RF, NB, etc.
Bhatt et al., 2020 [54]	Identified several factors associated with readmission after hospitalization; addressed conditions such as depression to help lower readmissions	Multivariable logistic regression
Rodríguez et al., 2020 [43]	Comprehensive study pertaining to Centers for Medicare & Medicaid Services State Innovation Models Critical outcome: greater investment in health information exchange and the intensive use of payment models that promote inter-organizational coordination	Multivariable logistic regression
Mourad et al., 2020 [55]	Focus group: women with diabetes and risk for 60-day postpartum readmissions and complications; efforts: coordinated care and close monitoring in the postpartum period. Research initiatives required to optimize postpartum follow-up with obstetric providers and endocrinologists	Log-linear regression models
Rodriguez-Gutierrez et al., 2019 [56]	Study of racial and ethnic differences in readmissions among US patients with diabetes; understanding the association of financial and hospital factors with readmission risk among racial groups	Logistic regression model
Hurtado et al., 2019 [57]	Investigating causes and predictors for 30-day readmissions for patients with diabetes ketoacidosis; identification of the most at-risk group: critical time window (within two weeks of discharge) for intervention	Multivariable regression model
Gregory et al., 2018 [44]	Comparison of different strategies associated with a low and high risk of readmissions for diabetes patients; the importance of diabetes survival skill education and medication reconciliation before discharge; scheduling follow-up phone calls after discharge and planning office visits to adjust diabetes regimen	Statistical analysis
Karunakaran et al., 2018 [58]	A comprehensive study of readmission risk factors based on predischarge and post-discharge data	Multivariable logistic regression
McCoy et al., 2017 [59]	Investigating common factors and reasons associated with severe dysglycemia for diabetes patients	Multivariable logistic regression
Chen et al., 2017 [45]	Examination of the association between preventable readmissions of patients with diabetes and ethnicity and neighborhood racial composition; emphasis on the need to include the factors of race/ethnicity and neighborhood racial composition in future models under the home health financial incentive program	Statistical analysis
Heaton et al., 2016 [60]	Comparison of risk for diabetes-related hospital readmissions for patients treated with sulfonylureas (SU) and other oral antihyperglycemic agents (AHAs)	Logistic regression model
Rubin et al., 2016 [61]	Identification of themes contributing to the risk of readmissions: health literacy; study of factors: following the discharge instructions, being aware of medication changes upon discharge, social support at home, social determinants of health impeding care, and loss of control over illness	Multivariable logistic regression
Raval et al., 2015 [62]	Retrospective assessment of hospital readmission rates and risk factors among elderly Medicare beneficiaries with type 2 DM.	Multivariable logistic regression

Characteristics of the Studies

The systematic review of the articles comprised an understanding of study designs, type(s) of DM addressed, independent and dependent variables, sample sizes, and databases accessed for analyses. These aspects of the selected studies specific to type 2 diabetes and overall DM are tabulated in Table 2. Overall, 10 out of 21 studies focused particularly on readmissions for type 2 DM patients, while six studies examined diabetes-related readmissions in general [42,44,46,58,59,61]. As shown in Table 3, four articles [49, 50, 52, 54] assessed readmissions for type 1 DM individuals, and one study [55] aimed at comparing 60-day readmissions for patients with type 1 DM, type 2 DM, and gestational diabetes. As depicted in Tables 2-3, the study designs comprised a retrospective cohort study (18 articles), one retrospective trend study [49], one prospective cohort study [44], and one quasi-experimental study [43]. Studies assessed readmissions through different datasets collected in the US over the years 1999-2018. The outcome (variable of interest) assessed in the studies was found to be "readmission," a time-dependent variable. Almost all articles used a 30-day readmission period, except for three articles, which had 60 days [55], 90 days [51], and one year [60] as time frames for the readmission studies. The sample size used for analysis varied from as low as 400 samples [48] to as large as 13,537,803 sample values [60].

**Table 2:** Characteristics of study design and predictor (independent) and outcome (dependent) variables for articles pertaining to diabetes mellitus (DM) and type 2 diabetes HbA1C: glycated hemoglobin; DSME: diabetes self-management education; LOS: length of stay; NA: not available

Author, year	Study design	Database	Sample size	Type(s) of diabetes mellitus (DM)	Readmission period (outcome)	Independent variables (number of variables)
Rubin et al., 2023 [47]	Retrospective cohort study	Patient discharge data Boston Medical Center 2004–2012	8,054	Type 2 DM	30 days	Sociodemographic, clinical, and administrative variables (49)
Timple and Kawar, 2022 [48]	Retrospective cohort study	Hospital clinical data: urban-managed hospital in Southern California in an integrated healthcare consortium	400	Type 2 DM	30 days	Patients’ demographics, hospital discharge disposition, HbA1C, medications, DSME, endocrinology consultation, LOS, and comorbidities (11)
Thyagaturu et al., 2021 [51]	Retrospective cohort study	Nationwide Readmissions Database (NRD) 2018	441,295	Type 2 DM	30 and 90 days	Patient demographics, insurance coverage, median household income, discharge disposition, comorbidities, severity of illness, hospital designation, bed size, and teaching status (NA)
Shang et al., 2021 [46]	Retrospective cohort study	Health Facts Database (Cerner Corporation, US) 1999–2008	100,244	DM	30 days	Race, sex, age, admission type, admission location, LOS, and drug use (23)
Smith et al., 2021 [53]	Retrospective cohort study	Medicare Beneficiary Summary File, Medicare Provider and Analysis Review, Home Health Outcome and Assessment Information Set, 2015	786,734	Type 2 DM	30 days	Patient demographics and clinical, geographic, neighborhood, and socioeconomic variables (NA)
Neto et al., 2021 [42]	Retrospective cohort study	Health Facts Database (Cerner Corporation, US) 1999–2008	101,766	DM	30 days	Attributes including race, sex, age, admission type, length of stay, admission location, and drug use (50)
Rodríguez et al., 2020 [43]	Quasi-experimental study	State Inpatient Databases (SID)* *2010–2015	3,391,599	Type 2 DM	30 days	Patient demographics, insurance coverage, comorbidities, and hospital designation (NA)
Rodriguez-Gutierrez et al., 2019 [56]	Retrospective cohort study	OptumLabs Data Warehouse 2009–2014	272,758	Type 2 DM	30 days	Demographic factors, clinical factors, economic factors, index hospitalization, and index hospital (NA)
Hurtado et al., 2019 [57]	Retrospective cohort study	NRD 2010–2014	479,590	Type 2 DM	30 days	Patient characteristics such as age, sex, median yearly income in the patient’s zip code, primary payer, relevant comorbidities, and comorbidity score (NA)
Gregory et al., 2018 [44]	Prospective cohort study	NewYork-Presbyterian (NYP)/Weill Cornell campus patient data 2014–2015	Varies	DM	30 days	HbA1c >9%, patients that were new to any insulin, or those intensified to a basal/bolus regimen (e.g., calculating bolus insulin based on blood glucose (BG) for the first time (NA)
Karunakaran et al., 2018 [58]	Retrospective cohort study	Boston Medical Center 2004–2012	17,284	DM	30 days	Hospital characteristics, sociodemographic, administrative factors, clinical measures (preadmission), etc. (48)
McCoy et al., 2017 [59]	Retrospective cohort study	OptumLabs Data Warehouse 2009–2014	594,146	DM	30 days	Patient characteristics, index hospital characteristics, and diabetes characteristics (NA)
Chen et al., 2017 [45]	Retrospective cohort study	Multiple 2009 data sources	N1 = 86,567 N2 = 17,262 N3 = 11,392	Type 2 DM	30 days	Preventable readmissions, predisposing factors, enabling factors, health conditions, functional status, organization characteristics, and community factors (NA)
Heaton et al., 2016 [60]	Retrospective cohort study	Medical Expenditure Panel Survey (MEPS) 1999–2010	13,537,803	Type 2 DM	1 year	Demographic characteristics, insurance coverage, period of first hospital admission, comorbidities, medical care received, and disease severity (NA)
Rubin et al., 2016 [61]	Retrospective cohort study	Patient discharge data Boston Medical Center 2004–2012	17,595	DM	30 days	Sociodemographic and hospital-related, diabetes-related, and other factors (46)
Raval et al., 2015 [62]	Retrospective cohort study	Humana Medicare Advantage with Prescription Drug (MAPD) database 2007–2012	202,496	Type 2 DM	30 days	Patients’ demographic, insurance, index hospital, and clinical characteristics; patient complexities specific to the elderly; and healthcare utilization (NA)

**Table 3:** Characteristics of study design and predictor (independent) and outcome (dependent) variables for articles pertaining to type 1 and gestational diabetes

Author, year	Study design	Database	Sample size	Type(s) of diabetes mellitus (DM)	Readmission period (outcome)	Independent variables (number of variables)
Shaka, 2022 [49]	Retrospective trend study	Nationwide Readmissions Database (NRD) 2010, 2012, 2014, 2016, 2018	Not available (NA)	Type 1 DM	30 days	Patient demographics, household income, primary payer, and comorbidity index (NA)
Vasireddy et al., 2021 [50]	Retrospective cohort study	NRD 2017	19,519	Type 1 DM	30 days	Patient demographics, insurance coverage, median household income, severity of illness, discharge disposition, comorbidities, hospital designation, and teaching status (NA)
Shaka et al., 2021 [52]	Retrospective cohort study	NRD 2018	94,668	Type 1 DM	30 days	Patient demographics, insurance coverage, median household income, comorbidities, hospital designation, bed size, and teaching status (NA)
Bhatt et al., 2020 [54]	Retrospective cohort study	NRD 2010–2014	4,055	Type 1 DM	30 days	Patient demographics, insurance coverage, median household income, severity of illness, discharge disposition, teaching status, comorbidities, hospital designation, and bed size (NA)
Mourad et al., 2020 [55]	Retrospective cohort study	NRD 2010–2014	248,241	Type 1 DM, Type 2 DM, Gestational diabetes, Unspecified diabetes	60 days	Patient demographics, clinical factors, and hospital characteristics (NA)

**Figure 2:**
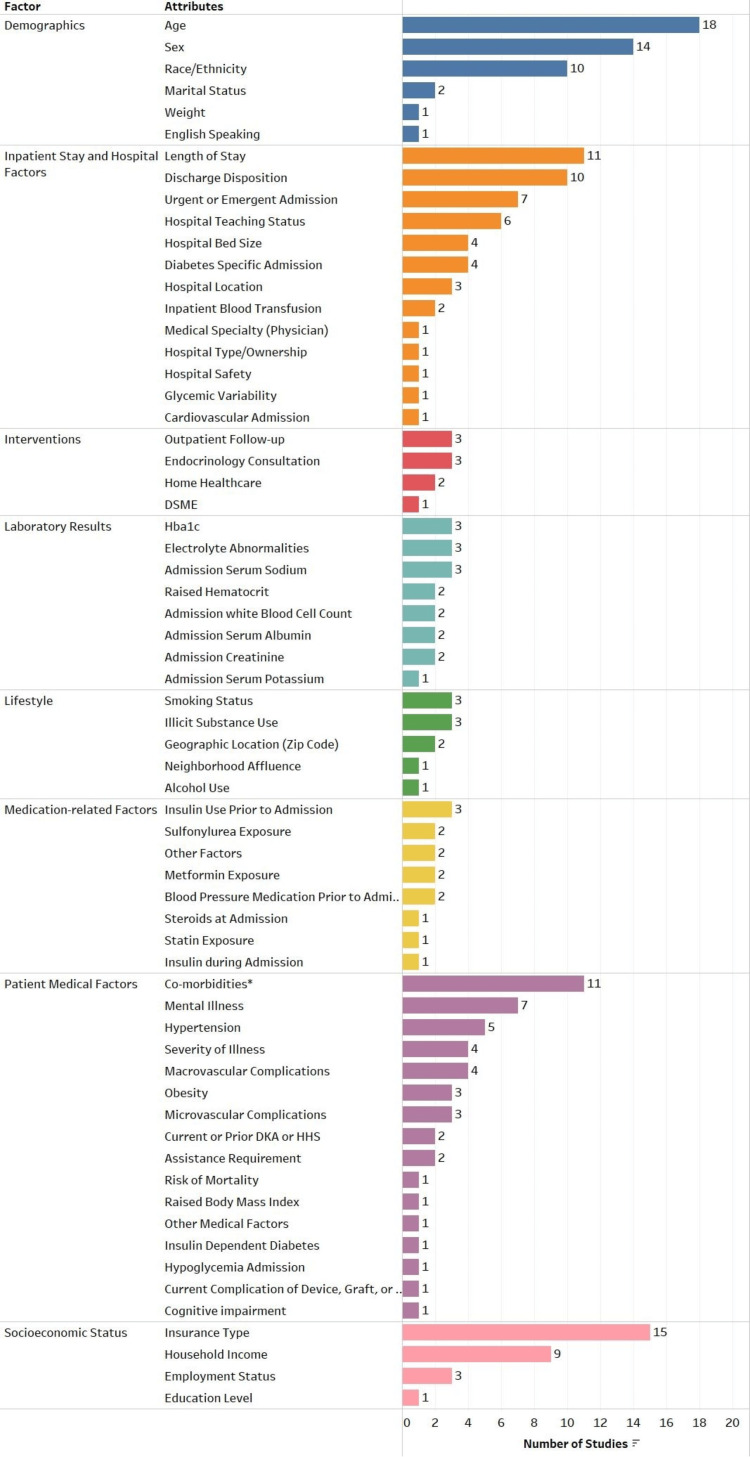
Summary of significant risk factors and interventions identified in the studies (*including CHF, cardiac dysrhythmias, COPD, liver disease, pancreatitis, etc.) CHF: congestive heart failure; COPD: chronic obstructive pulmonary disease; DSME: diabetes self-management education; HbA1C: glycated hemoglobin; DKA: diabetic ketoacidosis; HHS: hyperosmolar hyperglycemic syndrome This figure has been created by the authors.

Patient demographics, hospital characteristics, clinical attributes, geographical factors, and socioeconomic variables were reported as independent variables (predictors) in these studies. A detailed breakdown of significant risk factors and interventions identified in the reviewed articles is presented in Figure 2. The systematic review consisted of aggregating all risk factors responsible for diabetic readmissions across the studies. A total of 71 distinct risk factors were noted from the published studies after removing duplicates. These risk factors were found to be statistically significant in these research studies. Among the significant risk factors, patients’ age (18 studies), insurance type (15 studies), patients’ sex (14 studies), comorbidities (11 studies), length of stay (11 studies), discharge disposition (10 studies), race/ethnicity (10 studies), household income (nine studies), mental illness (seven studies), and type of admission (seven studies) were most identified for readmissions. In addition, four distinct interventional factors that impacted diabetes-related readmissions were also identified.

Meta-Analysis

A meta-analysis was conducted to quantitatively synthesize a few of the risk factors, including patient demographics and socioeconomic variables, to assess the likelihood of readmissions.

**Figure 3:**
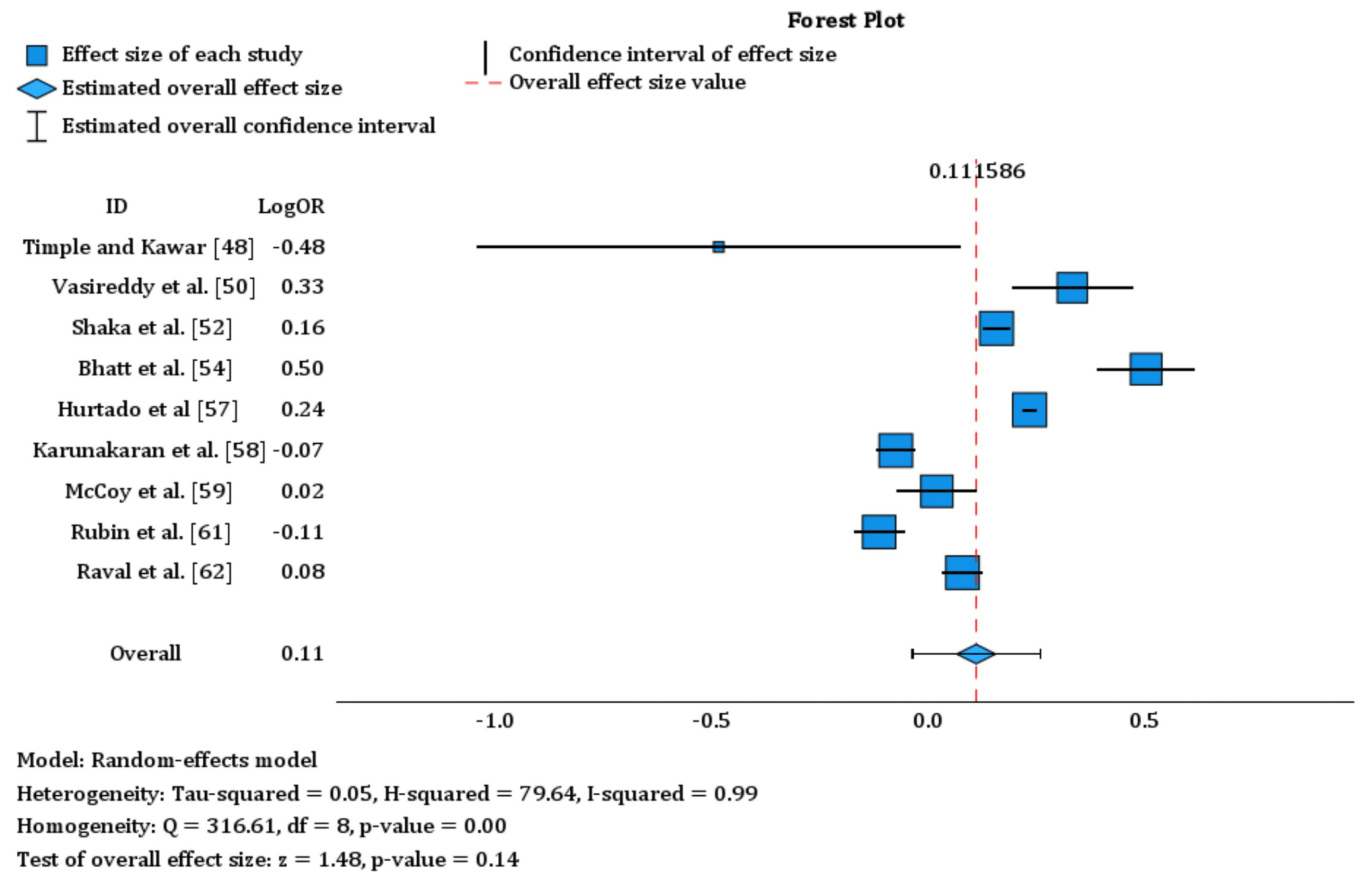
Forest plot of nine studies indicating sex as a risk factor for readmissions

Sex: The analysis of the effect of patients’ sex on 30-day readmissions involved nine studies [48,50,52,54,57-59,61,62] consisting of 734,755 males and 755,017 females. The forest plot of these studies considering patients’ sex as a risk factor is shown in Figure 3. The overall effect size estimate was 0.112 (standard error = 0.0754), yielding a p-value of 0.139 with a 95% confidence interval ranging from −0.036 to 0.259. The results indicated no statistical significance and a small effect size. The overall estimates indicated that females were found to have a slightly lower risk of readmissions compared to males, with an odds ratio (OR) of 0.11. The analysis revealed significant heterogeneity among the studies, with I-squared at 99%.

Race/ethnicity: The effect of race/ethnicity as a risk factor for readmissions was explored by using dichotomous categories: White patients compared to patients who identified as Black, Asian, Hispanic, and other racial/ethnic groups. The analysis was conducted on five studies [48,58,59,61,62], comprising a total of 578,344 White individuals and 289,311 individuals from other racial/ethnic groups. The effect size estimate for this analysis was -0.184, with a standard error of 0.0337. This resulted in a Z-score of −5.452, indicating statistical significance (p < 0.001). I-squared was found to be 0.78, indicating heterogeneity in effect sizes was not random.

Median household income: The meta-analysis included studies that reported median household income using four quartile ranges [50,52,54,57]. Income categories were combined into two dichotomous levels, with quartiles 1 and 2 in one group (low income consisting of 400,479 individuals) and quartiles 3 and 4 (high income consisting of 229,991 individuals) in another. The effect size estimate for the overall analysis was found to be 0.363, with a standard error of 0.0808. It resulted in a Z-score of 4.491 and indicated statistical significance (p <0.001). The 95% confidence interval ranged from 0.205 to 0.521, suggesting a significant effect of the low-income group over the high-income group for readmission risk.

Insurance type: The meta-analysis for insurance type included six studies [50,52,54,57,58,62], involving a total of 547,396 patients. Among these, 326,137 were enrolled in Medicare and/or Medicaid plans (group 1), while 221,259 were privately insured (group 2). Using these dichotomous groups, the analysis yielded an overall effect size of 0.783 (standard error = 0.1735) and a Z-score of 4.510, which demonstrated statistical significance (p <0.001). The results indicated that patients under Medicare and/or Medicaid plans had a higher risk of readmission as compared to privately insured persons. I-squared was found to be 1.00, demonstrating heterogeneity.

Assessment of Risk of Bias

**Figure 4:**
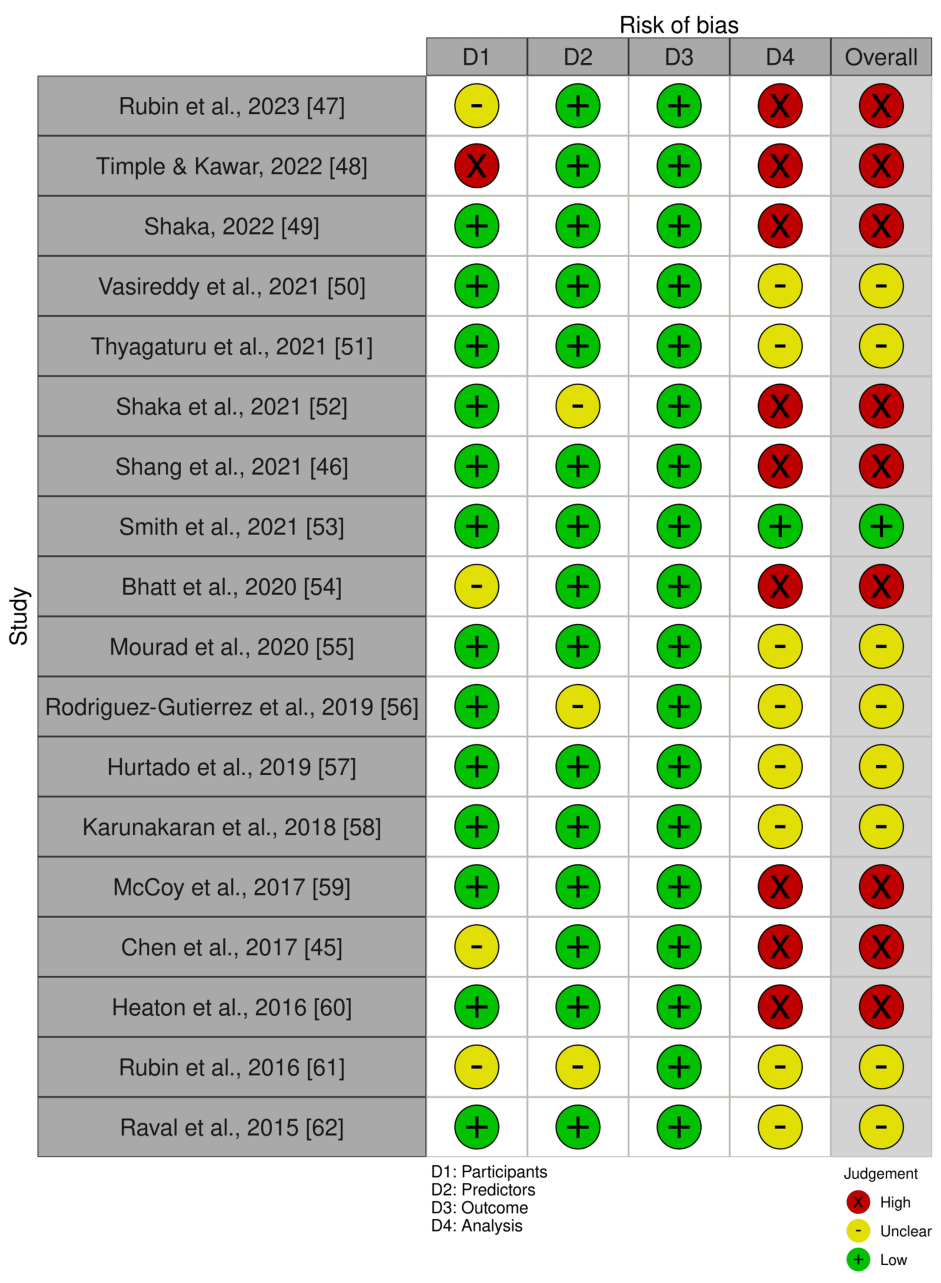
Risk of bias assessment for eighteen studies using Prediction Model Risk of Bias Assessment Tool (PROBAST)

**Figure 5:**
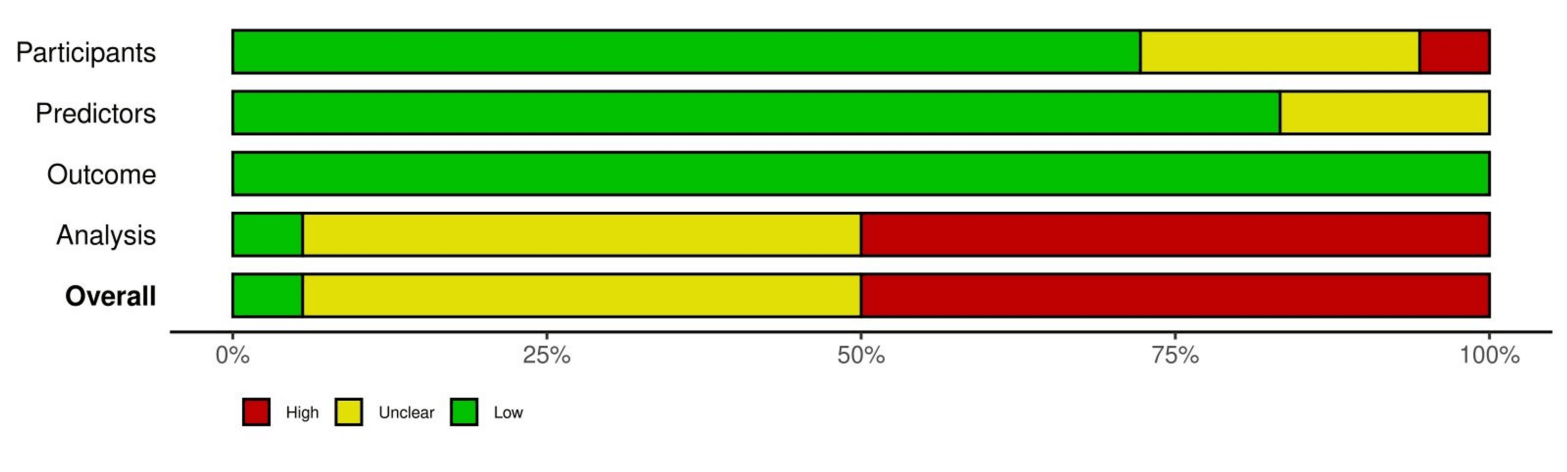
Summary of risk of bias for selected studies

Figure 4 shows the risk of bias assessment for 18 studies included in the analysis using PROBAST [41]. It was noted that the outcome domain demonstrated consistently low bias across all models, due to the retrospective nature of the studies and clarity of definition for “readmission” in the studies. It was observed that the selection of study participants exhibited some bias due to inclusion criteria specified in the various studies. With regard to independent (predictor) variables, the results showed a low bias. However, concerns regarding definitions of variables for certain articles resulted in the unclear risk of bias in this domain. The analysis domain displayed the highest risk of bias among all domains. This was mainly because of the inclusion of univariable analysis to select predictors, the conversion of continuous variables to dichotomous ones, and the handling of missing data. Overall, the quality of the studies was found to possess a relatively high risk of bias. Figure 5 presents the summary of the risk of bias for selected studies.

Discussion

Tables 4-5 present the significant findings of the studies included in this systematic review pertaining to DM, type 2 diabetes and type 1 DM, and gestational diabetes, respectively. Potentially preventable hospitalizations continue to be a major area of concern as they represent a huge financial burden across the healthcare ecosystem [58]. Various analytical tools, statistical methods, and data mining techniques hold the key potential to convert the overwhelming amount of electronic health records and hospital data into actionable knowledge. This aids in decision-making for improving care for diabetic patients and reducing readmission costs [42]. Characteristics of patients with higher readmissions included age, time of admission, diagnosis, number of emergencies, and gender [60]. Since elderly patients are at risk of a greater number of readmissions and emergency visits, a critical need for health-related education and follow-up to prevent complications was observed. Among the high-risk groups, chronic diseases, along with comorbidities and age, were the main factors for increased readmissions [47,62].

**Table 4:** Details of significant findings in the systematic review of studies pertaining to diabetes mellitus and type 2 diabetes

Author, year	Significant findings
Rubin et al., 2023 [47]	Diabetes patients with a primary discharge diagnosis have a higher readmission rate than diabetic patients with a secondary discharge diagnosis. Patients with a primary discharge diagnosis of diabetes were likely to have more extreme metabolic abnormalities. Inpatient consultation by a diabetes management service was associated with lower odds of readmission in people with a primary discharge diagnosis of diabetes.
Timple and Kawar, 2022 [48]	Hispanic patients with comorbidities receiving endocrinology consultations were more likely to be readmitted. Patients who received diabetes self-management education or prescribed both oral and insulin medications were less likely to be readmitted. Findings helped to empower nurse managers, leadership teams, and staff to implement targeted plans and evidence-based practice programs.
Thyagaturu et al., 2021 [51]	Patients with diabetes and heart failure showed an elevated risk of all-cause readmissions. Predictive models depict markers of lower socioeconomic status and the lowest quartile of median income as indicators of readmission.
Shang et al., 2021 [46]	Random forest (RF), naïve Bayes (NB), and tree ensemble methods resulted in an average area under the receiver operating characteristic (ROC) curve (AUC) of 0.661, 0.633, and 0.659 for 30-day readmissions. Findings emphasized education and follow-up for elderly patients with repeated admissions and machine learning (ML) techniques to reduce short-term readmissions.
Smith et al., 2021 [53]	For patients discharged to self-care, initiation of home healthcare on days three to seven was associated with 39% higher chances of rehospitalization compared to initiation of services in two days. Skilled home healthcare services should begin within 48 hours of referral or hospital discharge.
Neto et al., 2021 [42]	The best results for scenarios with all attributes after data preparation were used. The RF algorithm stands out with above 80% accuracy in four out of six scenarios independently of the sampling method.
Rodríguez et al., 2020 [43]	Lack of evidence towards reduction in readmissions through the Centers for Medicare & Medicaid Services (CMS) State Innovation Models (*SIM*) Initiative. Explicit examination of state-specific effects requires further empirical studies of the longer-term impact of SIM.
Rodriguez-Gutierrez et al., 2019 [56]	Individuals belonging to the Black community had a higher risk of readmission. A strong correlation was observed between lower income and patients hospitalized in non-profit, academic, or large hospitals was noted. Encouraged addressing reasons for persistent racial/ethnic health disparities.
Hurtado et al., 2019 [57]	Frequent readmissions for patients with end-stage renal disease, drug abuse, and discharge against medical advice. High-risk group patients readmitted within two weeks after discharge interventions are needed to reduce mortality, morbidity, and healthcare costs.
Gregory et al., 2018 [44]	Challenges in implementing comprehensive transitional care for diabetic high-risk patients. Interventions for preventing readmissions should be undertaken during hospitalization and continued after discharge.
Karunakaran et al., 2018 [58]	Noted risk factors such as outpatient visit absence after discharge, long hospital stays, and prior discharge within 90 days before the index admission. The strongest reasons were the employment status of the disabled, retired, or unemployed, race/ethnicity, health insurance coverage, comorbidity burden, and abnormal admission laboratory values.
McCoy et al., 2017 [59]	Findings showed that the hospitalization rate for severe dysglycemia is high. Diabetes-specific interventions may benefit younger patients and those with diabetes complications at the greatest risk of severe dysglycemia readmission.
Chen et al., 2017 [45]	Individuals from African American neighborhoods with moderate or high densities are at higher risk of readmissions. Differences in preventable readmissions between African American and White patients were attributed to disparities in the quality of care by health providers.
Heaton et al., 2016 [60]	The risk of readmission was 30% higher among type 2 diabetes mellitus (DM) patients who used SU than those using other antihyperglycemic agents (AHAs). Readmission costs on average for sulfonylureas (SU) users were 45% more than for those using other AHAs.
Rubin et al., 2016 [61]	Low socioeconomic status, racial/ethnic minorities, greater burden of comorbidities, public insurance, and recent hospitalizations contribute to readmission risk. Diabetes education, therapy, and outpatient diabetes care are more effective at reducing readmissions from all causes in patients with poorly controlled diabetes.
Raval et al., 2015 [62]	Patients with diabetes complications were more likely to be readmitted as compared to those without such complications. Importance of intervention programs for elderly patients with extensive complexities, suited to fit their needs.

**Table 5:** Details of significant findings in the systematic review of studies pertaining to type 1 diabetes mellitus and gestational diabetes

Author, year		Significant findings
Shaka, 2022 [49]	A significant disparity in outcomes among children with type 1 diabetes mellitus (DM). Longitudinal analysis confirms the persistence of this trend.	
Vasireddy et al., 2021 [50]	Lack of significant change observed in risk factors over the years 2001 to 2017. Comorbidities: depression and thyroid disorder had increased odds of 30-day readmission.	
Shaka et al., 2021 [52]	Predictors for readmission: hypertension, female sex, and discharge against medical advice. Findings reflect the need for implementing strategies to maximize adherence and compliance.	
Bhatt et al., 2020 [54]	Mental health illness, female sex, public insurance status, and discharge against medical advice are associated with increased readmissions. Initial hospitalization at a teaching hospital and private insurance related with decreased chances of readmission.	
Mourad et al., 2020 [55]	Women with diabetes are at increased risk of readmission 4.4% risk for women with type 1 DM, 3.9% for type 2 DM, and 2.0% for gestational diabetes. All-cause 60-day postpartum readmission is low. Findings suggest nursing support and management through a multi-disciplinary case approach.	

The high-risk nature of patients readmitted in the age group of 65 years or higher was attributed to endocrinology consultation rather than quality-of-care issues [48]. The authors emphasized the importance of continual diabetes self-management education (DSME) for patients with diabetic complications due to comorbidities that could act as a significant predictor of readmissions. Diabetes in combination with heart failure puts patients at increased risk for readmissions. The adverse effects of diabetes on the heart and comorbidity conditions were related to worse outcomes [47,51]. Based on patient characteristics of age and economic status, children from lower-income households had higher readmission risks [49]. It was noted that multidisciplinary teams in urban hospitals could help in reducing hospital readmissions. The caregivers faced challenges in managing diabetic conditions, such as monitoring diet and blood glucose and administering insulin to children with diabetes, which in turn demonstrates the need for targeted education for diabetes management and care in schools [50]. At home, the continual efforts of caregivers in providing healthcare reduced the risk of adverse events and facilitated a smooth transition back to normal life [53]. Moreover, it was reported that women with diabetes were at an increased risk of postpartum readmission, high maternal morbidity, and hypertensive diseases [55].

Additionally, patients belonging to Black ethnicity with lower incomes who were hospitalized in non-profit, academic, or large hospitals had a significantly higher risk of readmission [56]. Researchers also identified potentially modifiable risk factors, such as smoking and drug use, and outlined social disparities in readmission rates. Patients with private insurance were less likely to be readmitted within 30 days as compared to those with publicly funded insurance [57]. The importance of addressing depression as a risk factor for readmission after hospitalization was also noted as crucial for patients with diabetes [54]. Notably, the associated factors were found to be teenage, female, and discharge against medical advice. Hence, by utilizing prediction tools and early identification and management strategies, the economic burden of patients and readmission rates could be reduced, especially for this high-risk population. These discussions highlighted emerging themes, including the identification of demographic and clinical factors and the impact of comorbidities associated with increased risk of readmissions. Different disparities based on ethnicity, income, and healthcare access outlined the need for addressing social determinants of health. An emphasis on diabetes management and the significant role of caregivers and public health interventions also emerged as a crucial theme from the review. Finally, it was noted that the efficient utilization of prediction tools for the early identification of high-risk populations was identified to optimize healthcare outcomes.

Limitations

In the review, it was observed that the sampling methods used in the articles had limitations such as retrospective design, single-center data, and cross-sectional design. A large sample size, state-level links allowing for national estimates, and the exclusion of patients with comorbidities associated with index delivery hospitalization were some of the strengths of the studies. Although the results may be valid and generalizable, they may not be generalizable due to the limitations of administrative data, the lack of clinical details, the inability to track patients across years and individual states, and the inability to follow medication and individual management care plans. Despite conducting few meta-analyses, the diversity of readmission definitions and subsets of diabetes patients across studies precluded an extensive meta-analysis. We limited our research to US healthcare resources. It was noted that research from other countries and regions was needed to improve the overall global outcomes.

Practice implications

Home diabetes management continues to be a challenging task, as it requires individual commitment to health and priority to diabetes management. The daily regimen must change over time since diabetes is a chronic and progressive illness [44]. Research studies suggest that post-discharge measures needed interventions that should start during patient hospitalization to efficiently guide the patient for discharge and continue post-discharge to prevent readmissions. A reduction in readmissions and healthcare costs could be achieved by improving diabetes education, management, and medication adherence [63]. One of the policy implications suggested the crucial role of the hospital staff, such as nurses, in engaging in care coordination efforts. Assigning nurses to serve as diabetes champions in medicine units, ensuring that high-risk patients are given all the resources they need (e.g., referrals for additional education on diabetes), and regularly reviewing results for blood glucose monitoring were highlighted [44]. A multidisciplinary care intervention was required to improve outcomes in populations where sociodemographic factors played a key role in readmission risk [55].

Team efforts focused on developing specific admission protocols to meet the specific needs of diabetic patients could help prevent hospital readmissions [47,48]. Consequently, healthcare organizations should identify individuals leaving hospitals against medical advice and categorize them as high-risk groups using socioeconomic markers. The greater odds of inpatient mortality in readmissions due to inadequate treatment or shortened hospital stays signified a need for educating individuals and strict adherence to treatment plans [52]. Interventions targeting these patients could reduce readmissions and costs [51] and prevent associated morbidity and mortality [59]. In addition, the timely initiation of services, such as home healthcare, within 48 hours of hospital discharge for adult individuals with diabetes could help reduce readmission risks. Quality initiatives, such as referral and discharge plans, were necessary to be prioritized by healthcare providers and discharge planners [53].

Subsequently, patient characteristics, such as gender and age, should be prioritized as factors to develop interventions aimed at reducing readmissions. Healthcare providers and policymakers should provide access to quality care and reduce disparities related to health insurance coverage for lower-income communities, specifically to mitigate pediatric diabetes [49]. In school settings, policy implications to tackle pediatric diabetes, including coordinated post-discharge care using telemedicine, educating patients, families, and school staff about blood glucose monitoring, insulin dosage modulation, and related sick day supplies, were outlined [50]. The mental health issues that are associated with diabetes-related readmissions must also be addressed. Timely screenings for depression should be routine in high-risk patients, and interventions targeting its prevention and management should be incorporated into treatment plans [54].

The above discussion suggested that the reduction in ethnic disparity was possible by addressing neighborhood contextual factors through interventions [45]. The individuals belonging to African American communities who resided in high-density neighborhoods were at greater risk of 30-day preventable readmissions because for-profit home health providers were less likely to accept these patients. Significant racial/ethnic disparities in diabetes readmission rates cannot be explained alone by factors such as demographics, clinical factors, or socioeconomic status. Minority patients with diabetes may not be managed optimally in hospitals in deprived areas or in areas where community involvement is low. In addition, systemic biases and attitudes may result in inadequate patient support following hospitalization [56]. To reduce hospital readmissions at a population level, more investments must be made in health information exchanges [43]. Interorganizational coordination could be improved by employing health information exchange and more collaborative payment models. The healthcare industry must prioritize developing robust health information exchange systems and leveraging payment models that encourage collaborative care across multiple organizations.

## Conclusions

Hospital readmissions for diabetic patients pose a global concern that has shown escalating trends over the past decade. They continue to be a significant financial burden on the healthcare system and associated stakeholders. In this comprehensive review, recent research articles were studied to understand the key risk factors and characteristics pertaining to readmissions of diabetes patients. Using the PRISMA framework, 21 articles were identified and analyzed. These findings highlighted the importance of age, demographics, income, insurance policies, severity of illness, and comorbidities as critical factors. Through this study, socioeconomic status, health conditions, and hospital care quality were found to be the main contributors to readmissions. Emergent themes from the findings suggested that timely screenings and lifestyle changes could reduce diabetes-related complications and preventable hospitalizations, improving healthcare delivery. Specific strategies related to diabetes education, interventions, and interdisciplinary team efforts could help to prevent readmission risks. The study also showed that it was necessary to conduct large, randomized trials to assess the effectiveness of these policies and quality initiatives.
